# From bench to clinic: the development of VLA1553/IXCHIQ, a live-attenuated chikungunya vaccine

**DOI:** 10.1093/jtm/taae123

**Published:** 2024-09-10

**Authors:** Lin H Chen, Andrea Fritzer, Romana Hochreiter, Katrin Dubischar, Stéphanie Meyer

**Affiliations:** Department of Medicine, Division of Infectious Diseases and Travel Medicine, Mount Auburn Hospital, 330 Mt Auburn St, Cambridge, MA 02138, USA; Faculty of Medicine, Harvard Medical School, 25 Shattuck St, Boston, MA 02115, USA; Pre-Clinical Vaccine Development Department, Valneva Austria GmbH, Campus-Vienna-Biocenter 3, 1030 Vienna, Austria; Clinical Serology Department, Valneva Austria GmbH, Campus-Vienna-Biocenter 3, 1030 Vienna, Austria; R&D Management, Valneva Austria GmbH, Campus-Vienna-Biocenter 3, 1030 Vienna, Austria; Corporate Medical Affairs, Valneva SE, Ilot Saint-Joseph Bureaux Convergence, 12 ter Quai Perrache Bâtiment A, 69002 Lyon, France

**Keywords:** chikungunya, arbovirus, vaccine, immunogenicity, safety, surrogate of protection, VLA1553

## Abstract

**Background:**

Over the past 20 years, over 5 million cases of chikungunya, a mosquito-transmitted viral disease, have been reported in over 110 countries. Until recently, preventative strategies for chikungunya were largely ineffective, relying on vector control and individual avoidance of mosquito bites.

**Methods:**

This review outlines the preclinical and clinical efficacy and safety data that led to the approval of VLA1553 (IXCHIQ^®^), a live-attenuated vaccine against chikungunya disease. It also describes the innovative development pathway of VLA1553, based on an immunological surrogate of protection, and discusses ongoing and future post-licensure studies.

**Results:**

In mice and non-human primate models, VLA1553 elicited high titres of neutralizing antibodies, conferred protection against wild-type chikungunya virus challenge and raised no safety concerns. A Phase 1 clinical trial of VLA1553 demonstrated 100% seroconversion among 120 healthy participants, with sustained neutralizing antibody titres after 12 months. These results and determination of a surrogate marker of protection led to advancement of VLA1553 directly into Phase 3 clinical development, as agreed with the US Food and Drug Administration (FDA) and the European Medicines Agency. The pivotal Phase 3 trial met its primary immunogenicity endpoint, achieving seroprotective levels based on immuno-bridging in baseline seronegative participants 28 days post-vaccination. These findings enabled submission of a Biologics Licence Application to the FDA for accelerated approval of VLA1553 in the US for adults aged ≥18 years. Ongoing and planned studies will confirm the clinical efficacy/effectiveness and safety of VLA1553 in adults and younger individuals, and will generate data in chikungunya endemic countries that have the highest unmet need.

**Conclusion:**

VLA1553 is the first vaccine approved for the prevention of chikungunya disease in adults, following accelerated development based on a serological surrogate marker of protection. VLA1553 adds to strategies to reduce the spread and burden of chikungunya in endemic populations and travellers.

## Introduction

Chikungunya, a mosquito-borne viral disease caused by the chikungunya virus (CHIKV), is transmitted to humans by *Aedes* mosquitoes, primarily *Aedes aegypti* and *Aedes albopictus*.^1–4^ Over the last two decades, over 5 million chikungunya cases have been reported in over 110 countries across Africa, Asia, South America and more temperate regions of Europe and North America.^2–12^ The changing climate, urbanization, rapid pace of globalization and remarkable resilience of *Aedes* mosquitoes may drive the spread of habitats for *A. aegypti* and *A. albopictus* even further.^13–15^ Together with increased fitness of transmission by *A. albopictus* following a viral mutation, this escalates the threat of chikungunya outbreaks and increased disease circulation globally.^13^^,^^14^^,^^16^

Clinical manifestations range from asymptomatic infection in a minority of patients to severe debilitating illness that may last years.^17–20^ Up to 75% of CHIKV-infected patients are symptomatic with fever, headache, nausea, fatigue, skin rash, myalgia or polyarthralgia;^1^^,^^17^^,^^18^^,^^21–23^ an estimated 43% experience chronic symptoms, with a mortality rate of 3 in 1,000 cases.^18–20^^,^^22^ CHIKV infection impacts quality of life significantly and is associated with substantial psychosocial and economic burden.^21^^,^^24–27^ For example, the total estimated cost associated with the 2014–15 chikungunya outbreak in the US Virgin Islands ranged from $14.8 to $33.4 million, approximately 1% of gross domestic product.^21^ Further research is needed on the economic impact of travel-related chikungunya.^28^

Since its discovery in Tanzania in 1953, CHIKV has been responsible for periodic outbreaks in several African countries, eventually spreading to the Asian subcontinent and the Americas via the Caribbean.^23^^,^^29^ CHIKV is classified into three main genetically distinct lineages based on the region of evolution: West African, East-Central-South African (ECSA) and Asian;^23^ a mutation in ECSA glycoprotein during the 2005–06 outbreaks derived the Indian Ocean lineage (IOL) that adapted to *A. albopictus* and enhanced viral transmission.^3^^,^^6^ The ECSA lineage is associated with outbreaks in Africa, Asia and Europe and some in the northeast region of Brazil. The Asian lineage is mainly associated with outbreaks in Latin America.^11^

International air travel has greatly facilitated the spread of CHIKV, and CHIKV circulation is sometimes recognized by returning travellers or years later through retrospective serosurveys.^30^^,^^31^ An analysis of travel-related confirmed or probable chikungunya reported to GeoSentinel from 2005–20 identified almost 100 destinations globally where international travellers had CHIKV-exposure.^32^ Travellers should be aware of the potential risk of CHIKV infection, the risk of chronic rheumatologic sequelae, and the importance of seeking medical attention.^23^

Chikungunya outbreaks are sporadic and unpredictable. The most recent large-scale outbreak of CHIKV was reported in Paraguay.^11^^,^^33^ Between October 2022 and April 2023, 118,179 suspected and confirmed chikungunya cases were recorded by the Ministry of Health.^34^ Overall, 46 deaths and 125 suspected cases of CHIKV-related acute meningoencephalitis were reported.^34^ Of cases reported between October 1 2022 and March 11 2023, 94% occurred during 2023, leading to an epidemiological alert issued by the Pan American Health Organization and World Health Organization that member states prepare healthcare services for the possible spread and potential outbreak of CHIKV. This was the third outbreak reported in Paraguay since 2015.^33^^,^^35^^,^^36^

Until recently, prevention of chikungunya relied on vector control and avoidance of mosquito bites with unclear effectiveness.^37^^,^^38^ Vaccines have proved to be the most effective way to prevent infectious diseases. An effective vaccine will decrease disease burden, chikungunya-associated chronic morbidity and economic losses, especially in low- and middle-income countries and socio-economically deprived areas with higher incidences of mosquito-borne diseases.^39^ However, for emerging and re-emerging epidemic pathogens, challenges in conducting randomized controlled trials include unpredictable timing, location, size and duration of outbreaks and—if affecting primarily resource-limited countries—poor infrastructure for trial execution.^40^

An accelerated development pathway has the potential to overcome these challenges, thereby facilitating early access and supply of a licensed vaccine to minimize the spread and burden of disease, and prevent or rapidly curb future outbreaks. This approach was used in the development of VLA1553 (IXCHIQ^®^), the first chikungunya vaccine approved by the US Food and Drug Administration (FDA) in November 2023 for adults who are at increased risk of CHIKV exposure.^41^ The FDA decision was based on pivotal Phase 3 data showing that a single vaccination with VLA1553, a live-attenuated vaccine, generated a strong immune response and seroprotective titres in 98.9% of the vaccinated participants.^42^ Here we review the preclinical and clinical data, highlighting the innovative accelerated pathway, that led to the approval of VLA1553. Ongoing and future post-licensure studies are also described.

### Preclinical evaluation of VLA1553

VLA1553 is based on the La Réunion (LR)2006-OPY1 CHIKV infectious clone. This ECSA-IOL strain was attenuated to generate a genetically stable vaccine, VLA1553.^43^ In a mouse model, a single dose of VLA1553 induced potent humoral and cellular immunity against CHIKV infection.^43^ In cynomolgus macaques, pathogenesis of CHIKV infection mirrors human disease, providing robust endpoints for efficacy testing. A single vaccination with VLA1553 generated high titres of neutralizing antibodies that persisted for months after immunization and conferred protection from wild-type CHIKV challenge.^44^ VLA1553 proved to be safe, and the vaccinated animals did not show any CHIKV-associated findings such as fever, lymphopenia, rash or joint swelling in contrast to wild-type CHIKV-infected animals.^44^ Antibody responses induced by VLA1553 showed cross-neutralization, as VLA1553, based on an ECSA-IOL strain, generated neutralizing antibodies against a Caribbean strain of CHIKV.^44^

### Phase 1: randomized controlled trial in healthy adults^45^

A randomized, observer-blind, dose-escalation Phase 1 trial (NCT03382964) assessed the safety and immunogenicity of VLA1553 in 120 healthy adults in the US (aged 18–45 years). Participants were randomly assigned (1:1:2) to receive one of three escalating VLA1553 doses (3.2 × 10^3^ per 0.1 mL; 3.2 × 10^4^ per 1 mL; or 3.2 × 10^5^, 50% tissue culture infectious dose [TCID_50_] per 1 mL). Serum samples were analysed using the micro-neutralization (μNT_50_) assay, defined as a 50% reduction of cytopathic effect, to determine antibody kinetics and immunogenicity. Seroconversion was defined as the proportion of subjects achieving a CHIKV-specific neutralizing antibody titre of μNT_50_ ≥ 20. VLA1553 was well-tolerated and immunogenic in all dose groups after a single vaccination. In all three groups, viraemia peaked at Day 3 and resolved by Day 14, with seroconversion rates of 100% achieved by Day 14 and sustained for 1 year. After re-vaccination, participants did not experience vaccine-induced viraemia, indicating presence of neutralizing antibodies. Since antibody levels reached a plateau in all groups after a single vaccination, and re-vaccination did not further increase geometric mean titres (GMTs), no further dosing schedules were investigated. The medium dose of VLA1553 was selected to proceed directly to Phase 3 testing.^42^

### Serological surrogate endpoint determination^46^

A surrogate is defined as a ‘marker, such as a laboratory measurement, radiographic image, physical sign or other measure that is thought to predict clinical benefit but is not itself a measure of clinical benefit’.^47^^,^^48^ To establish a serological surrogate of protection for VLA1553, data were derived from an NHP passive transfer study^46^ and a sero-epidemiological study.^49^ Serum from VLA1553-vaccinated human participants of the Phase 1 study was transferred at varying titre levels to NHPs, which were then challenged with wild-type CHIKV LR2006-OPY1 1 day later. Pre-challenge serum samples from NHPs that had received different human serum pools were analysed using μPRNT. The surrogate of protection was based on protection from viraemia (undetectable CHIKV RNA in plasma) and absence of fever. Further details can be found in the supplement (Supplementary Figure 1).

**Figure 1:**
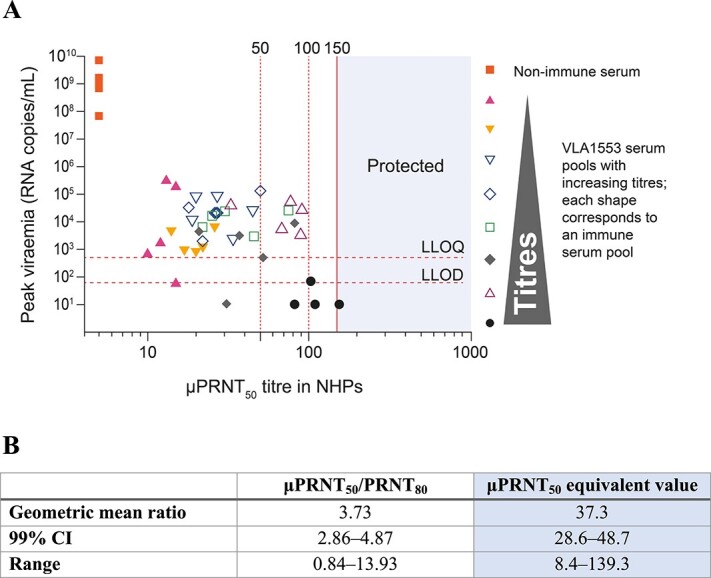
Surrogate of protection determination by analysing samples from NHP passive transfer study^46^ (A) and confirmation of surrogate of protection by analysing samples from Yoon *et al*.^49^ (B). A. Peak viraemia titres plotted against μPRNT_50_ titres from the NHP passive transfer study. Horizontal dotted lines show LLOQ (500 copies/mL) and LLOD (60 copies/mL) of the qPCR. Vertical dotted lines show μPRNT_50_ titres of 50 and 100, while the vertical solid line μPRNT_50_ titre of 150. B. Comparison of neutralization antibody titre results measured by μPRNT_50_ assay or reported by PRNT_80_ assay (Yoon *et al*.^49^). CI, confidence interval; LLOD, lower limit of detection; LLOQ, lower limit of quantification; NHP, non-human primate; PRNT, plaque reduction neutralization test; PRNT_80_, neutralization titre using an 80% plaque reduction; qPCR, quantitative polymerase chain reaction; μPRNT_50_, neutralization titre determined in a micro-neutralization assay (96 well format) using a 50% plaque reduction. Figure 1A is adapted from^46^ under the terms of the Creative Commons Attribution 4.0 International Licence (https://creativecommons.org/licenses/by/4.0/deed.en).

The threshold titre for protection, at which none of the animals had detectable CHIKV RNA, was set at a μPRNT_50_ titre of ≥150 (Figure 1A). This surrogate was conservative as it is considerably higher than the titre required to protect replicating CHIKV using a TCID_50_ assay and to protect animals from clinical signs of chikungunya disease such as fever.

To provide a second line of evidence to support the protective antibody threshold of μPRNT_50_ ≥ 150, sera from a study conducted in the Philippines were analysed.^49^ A sero-epidemiological study (*n =* 853) by Yoon *et al*. showed that participants with a positive neutralization titre of PRNT_80_ ≥ 10 were protected 100% (95% confidence interval [CI], 46.1–100.0) from symptomatic CHIKV infection.^49^ Sera from 33 subjects in the Yoon *et al*. study were tested in the μPRNT_50_ assay used for the immunogenicity analysis of VLA1553 to determine how the PRNT_80_ threshold of 10 compares to the μPRNT_50_ threshold of 150. The neutralizing antibody titre showed a correlation between both testing methods (Figure 1B). Further details can be found in the supplement.

The FDA agreed with the μPRNT_50_ titre of ≥150 as the definition of seroresponse and as a surrogate endpoint. Due to the unpredictability and transient nature of chikungunya outbreaks, it is challenging to perform Phase 3 efficacy trials and defining this surrogate of protection was an innovative and necessary step towards approval of the vaccine without efficacy data, as recommended by the FDA and European Medicines Agency (EMA).^50^^,^^51^

### Pivotal Phase 3 trial of VLA1553^42^

This randomized, placebo-controlled, double-blind, pivotal, Phase 3 trial (NCT04546724) aimed to assess the safety and immunogenicity of a single VLA1553 vaccination up to 180 days post-vaccination. A total of 4128 healthy adults (aged ≥18 years) from 43 sites within the US were randomized to receive VLA1553 (1 × 10^4^ TCID_50_ in 0.5 mL; *n =* 3093) or placebo (*n =* 1035), stratified by age. The primary endpoint was the proportion of baseline-negative participants with a seroresponse, defined as a CHIKV-specific neutralizing antibody titre of ≥150 in the μPRNT_50_ assay, at 28 days post-vaccination. The immunogenicity analysis comprised 362 participants (266 in the VLA1553 group and 96 in the placebo group).

**Figure 2:**
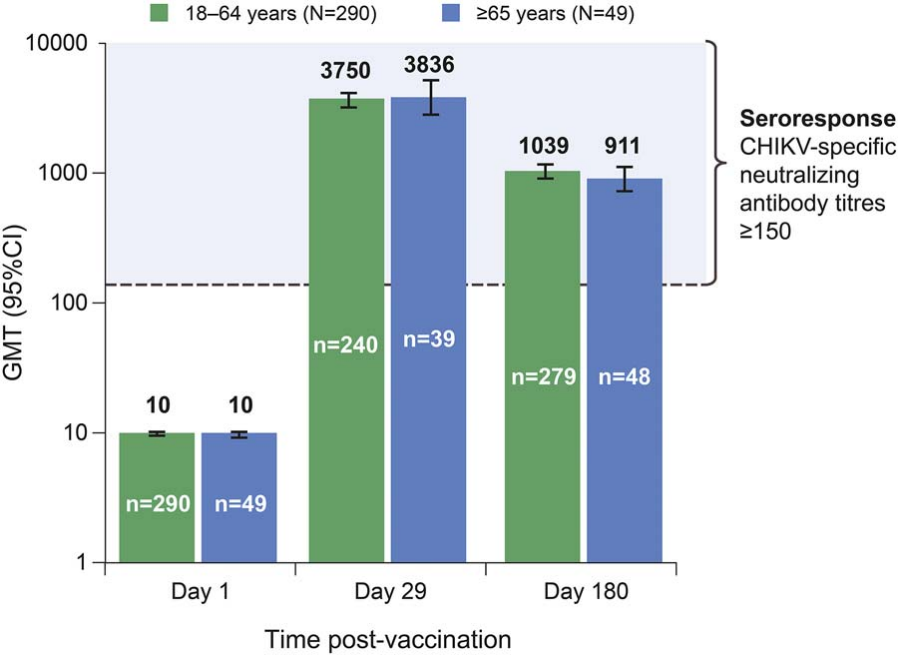
Assessment of CHIKV-specific neutralizing antibodies GMTs after vaccination, stratified by study day and age from the pivotal Phase 3 trial (per protocol population). Days shown in the figure refer to study days; Day 1 = day of vaccination. Error bars indicate 95% CIs. Neutralizing antibodies to the vaccine were evaluated from clinical specimen (human serum) using μPRNT_50_. CI, confidence interval; GMT, geometric mean titre; μPRNT_50_; neutralization titre determined in a micro-neutralization assay (96 well format) using a 50% plaque reduction.

After a single vaccination, VLA1553 induced seroresponse in 98.9% of participants by Day 28, independent of age. This exceeded the non-acceptance threshold of 70% of participants for the primary endpoint. The seroconversion rate was 99.2% on Day 29 and remained stable throughout the trial, with 98.3% of the participants maintaining seroconversion 6 months post-vaccination (Figure 2). High seroresponse rates were sustained 6 months post-vaccination in 96.3% of participants. Immune responses were similar in participants aged ≥65 years and <65 years over the duration of the trial.

VLA1553 was generally well tolerated across age groups and the majority of adverse events (AEs) were mild or moderate. Serious AEs (SAEs) were reported in 1.5% of participants and treatment-related SAEs in 0.1% of participants receiving VLA1553. Solicited systemic AEs within 10 days post-vaccination occurred in ~ 50% of VLA1553 versus ~ 27% of placebo recipients;^52^ the most commonly reported of these were headache (31.6%), fatigue (28.5%), myalgia (23.9%), arthralgia (17.2%), fever (13.5%) and nausea (11.2%).^53^ Any related severe systemic AEs occurred in 2% of VLA1553 versus 0.1% of placebo recipients;^52^ the most common being fever (1.4%), arthralgia (0.3%), myalgia (0.3%).^53^ Safety profiles were similar in participants aged ≥65 years and <65 years. Signs and symptoms, indicative of an acute, potentially vaccine-related CHIKV infection, were closely monitored as AEs of special interest (AESIs). AESIs were reported in 0.3% (10/3082) and 0.1% (1/1033) of participants in the VLA1553 group and the placebo group, respectively. Most AESI events were self-limiting, resolving after 2–4 days.

Following the trial, regulators requested a retrospective analysis of AESIs using a broader and less specific definition (Table 1) than the one previously used.^54^ The majority of chikungunya-like AEs under this broad definition were also seen with other licensed, highly immunogenic vaccines.^53^^,^^55–57^ Based on this, regulators considered that a warning should be added to the label due to the possibility of severe or prolonged reactions.^54^ In the pivotal Phase 3 trial, chikungunya-like AEs occurred in 11.7% (361/3082) of vaccine recipients and 0.6% (6/1033) of placebo recipients.^53^ Severe chikungunya-like AEs and prolonged AEs that lasted ≥30 days occurred in 1.6% (48/3082) and in 0.5% (14/3082) of vaccine recipients, respectively, with none reported by placebo recipients.^53^

**Table 1:** Chikungunya-like AEs^53^

Chikungunya-like AEs
• Fever and • ≥1 of any of the following: $\quad\circ $ arthralgia or arthritis, $\quad\circ $ myalgia, $\quad\circ $ headache, $\quad\circ $ back pain, $\quad\circ $ rash, $\quad\circ $ lymphadenopathy, or certain neurological, cardiac or ocular symptoms that occurred within 30 days after vaccination

AE, adverse event.

The strong immune response, acceptable safety profile and generation of seroresponse in nearly 100% of vaccinated participants, independent of age, well positioned VLA1553 as a candidate for the first approved vaccine for active immunization and prevention of chikungunya. Although the primary public health target is endemic areas, the first licensure step was in the US in CHIK-naïve populations who will likely benefit from VLA1553 as a travellers vaccine.

### Re-testing of Phase 1 sera using the Phase 3 μPRNT assay^58^

In the Phase 1 trial, VLA1553 was administered as a liquid frozen drug product, whereas for the subsequent clinical trials, a lyophilized drug product with an improved stability profile was administered. The immunogenicity of the frozen and lyophilized formulations of VLA1553 were compared using the validated μPRNT assay from the serological endpoint determination study.^46^ Sera were collected at Days 14, 28, 84, 180 and 365 post-vaccination. At 14 days post-vaccination, high GMTs were already reached (range of μPRNT_50_, 1815–3013; *n =* 90 participants tested) and peaked at Day 28 post-vaccination (range of μPRNT_50_, 4209–5034). GMTs decreased but remained at high levels through Day 365 (range of μPRNT_50_, 851–1138; *n =* 91 participants tested). All participants (*n =* 120) achieved seroresponse by Day 14 (100% across all groups), and 119 had sustained seroresponses at Day 365 (range 98–100%). Results from re-testing sera confirmed the initial Phase 1 analysis. The tested doses were highly immunogenic and seroresponses persisted for 1 year.

### Lot-to-lot consistency of VLA1553^59^

This prospective, randomized, double-blind, Phase 3 study (NCT04786444) evaluated VLA1553 in healthy adult participants (*n =* 408) who were equally randomized to one of three vaccine lots. The primary endpoint was a comparison of the GMT ratios of CHIKV-specific neutralizing antibodies between the three VLA1553 lots at 28 days post-vaccination. Secondary endpoints included comparison of the GMT ratios, immunogenicity and safety investigations over 6 months post-vaccination.

Seroresponse was achieved in 97.8% of participants on Day 29 and was maintained in 96.0% of participants at 180 days post-vaccination, irrespective of the lot used. Overall, AEs reported were mostly mild or moderate, with only 3.9% of participants experiencing severe AEs. There were no significant differences in the overall AE type or frequency between the lots, further substantiating the findings of the pivotal Phase 3 study.^42^

At 28 days post-vaccination, the three VLA1553 lots elicited comparable neutralizing antibody titres, demonstrating the consistency of the manufacturing process.

### VLA1553 demonstrated a broad spectrum of neutralizing antibody activity against all major CHIKV genotypes and closely related alphaviruses^60^^,^^61^

A panel of human sera collected 28 days post-vaccination with VLA1553 from a Phase 3 clinical study showed neutralization against wild-type CHIKV strains from different lineages, including the LR strain (ECSA-IOL), strain 37 997 (West African lineage), and the Caribbean M109 strain (Asian lineage) in PRNT assays. Sustained neutralization was seen with sera collected on Days 85 and 180 post-immunization.^60^ Another analysis of human post-vaccination sera (*n =* 30) characterized neutralizing capacity against LR2006-OPY1 (ECSA genotype), 181/25 (Asian genotype), and Brazil 7124 (2021 isolate from Tocantins, Brazil of the ECSA genotype) using PRNT assays. VLA1553 induced neutralizing antibodies against all CHIKV genotypes in all sera (*n =* 30/30) tested at similar levels, with a peak titre at Day 29 and persistently high levels 1-year post-vaccination.^61^ In addition, the cross-neutralizing potential extended to other related alphaviruses such as O’nyong-nyong, Mayaro, and to a lesser degree to Ross River.

### Regulatory aspects of VLA1553 vaccine development pathway

As described above, an accelerated pathway approach was used in the development of VLA1553, based on a surrogate marker. This follows the guidance in the FDA Safety Innovations Act, passed by the US Congress in 2012, which allows the FDA to accelerate approval for pharmaceutical candidates for serious conditions that fill an unmet medical need based on a surrogate endpoint.^50^

**Figure 3:**
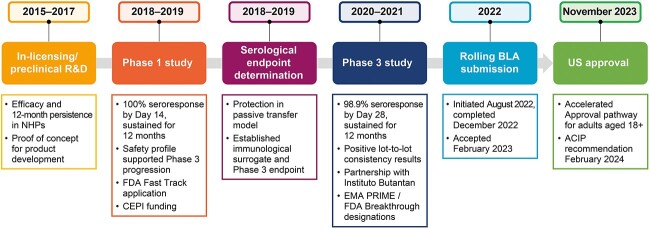
VLA1553 development pathway. ACIP, Advisory committee on immunization practices; BLA, Biologics Licence Application; CEPI, Coalition for Epidemic Preparedness Innovations; EMA, European Medicines Agency; FDA, US Food and Drug Administration; NHP, non-human primate; PRIME, PRIority Medicines; R&D, research and development.

To gain regulatory support for a novel vaccine approval pathway, VLA1553 was first granted ‘fast track’ designation by the FDA/Center for Biologics Evaluation and Research in 2018, a status that is intended to accelerate availability of promising medicinal products (Figure 3).^62^ The FDA’s Vaccines and Related Biological Products Advisory Committee (VRBPAC) endorsed chikungunya vaccine candidates to be licensed under the FDA’s ‘accelerated approval’ pathway, whereby a product can be licensed after it demonstrates benefit on a surrogate endpoint that is considered ‘reasonably likely’ to predict clinical benefit. In November 2019, the VRBPAC played a key role in enabling this licensure pathway for VLA1553.^63^ An NHP model of CHIKV infection and disease was agreed upon with the FDA to establish a surrogate of protection that supported regulatory conditions for licensure under the accelerated approval pathway. The EMA endorsed the approach and considered chikungunya vaccines approvable on the basis of immunological markers, with emphasis given to support for a surrogate of protection through analyses of the sero-epidemiological study performed in the Philippines.^49^ The Phase 1 clinical trial of VLA1553 resulted in 100% seroconversion of the 120 healthy participants, and antibody titres were sustained after 12 months. Based on these results, and in agreement with the regulatory bodies, VLA1553 advanced directly into Phase 3 clinical development. The pivotal Phase 3 clinical trial of VLA1553 met the primary endpoint, which was the proportion of patients with a seroresponse above the level of ‘surrogate of protection’ agreed with the FDA, at 28 days post-vaccination in baseline seronegative participants. The results of this pivotal study formed the basis of the Biologics Licence Application to the FDA for approval of the vaccine for use in adults aged ≥18 years. VLA1553 received FDA Breakthrough Designation in 2021 and was granted PRIority MEdicine designation and accelerated assessment by the EMA in 2020 and 2023, respectively.^64^ In May 2024, the Committee for Medicinal Products for Human Use of the EMA adopted a positive opinion recommending authorization of VLA1553 for the prevention of disease caused by CHIKV in individuals aged ≥18 years, and in June 2024, Health Canada approved the vaccine for use in the same population.^65^^,^^66^ Evidence is promising, suggesting that single immunization with VLA1553 provides rapid and long-term protection against CHIKV, and is intended to protect those living in or travelling to at-risk areas.^59^^,^^67^

### Future development

The US Centers for Disease Control and Prevention (CDC) defines ‘areas at risk for chikungunya’ if there has been outbreaks or reported cases within the last 5-year period. Therefore, countries such as Puerto Rico that were high risk in 2015–16 and have had no laboratory-confirmed symptomatic cases since 2017 is currently not defined as a high-risk area.^8^^,^^68^ However, the disease burden of CHIKV may be higher than described due to inconsistencies in diagnostics and likely under-reporting in endemic areas.^19^^,^^69^ Although the rate at which susceptible individuals acquire CHIKV is lower in endemic versus epidemic countries, large, unpredictable and sporadic outbreaks can escalate to give a long-term cumulative incidence of chikungunya that is equivalent to an epidemic.^70^ Notably, in 2023, the absolute number of positive tests for CHIKV was 3.8 times higher than for dengue in the Brazilian city of Belo Horizonte.^71^ Therefore, travellers (especially at-risk travellers to endemic regions with no recent outbreak but with a history of CHIKV transmission) should continue to take preventative measures.^72^

Under the accelerated approval pathway, confirmatory post-licensure trials demonstrating vaccine efficacy and safety are required. No safety concerns have been identified across all trials of VLA1553 by an independent data safety monitoring board, which reviews accruing safety data. Multiple studies are ongoing or planned to support the efficacy and safety of VLA1553 in adults and younger individuals as well as to generate data in endemic countries to enable vaccine access in high-demand areas. As there is a paucity of understanding of VLA1553 in humans, long-term assessment in wider populations, including immunocompromised patients and pregnant women, is integral.

### Post-licensure studies in the wider population

#### VLA1553 long-term study

An open-label, Phase 3b, single-arm study (NCT04838444) is evaluating the persistence of antibodies and long-term safety in 363 participants rolled over from the pivotal Phase 3 study. Subjects will have annual follow-up visits at Months 12, 24, 36, 48 and 60 post-vaccination. The primary objective is to evaluate the persistence of antibodies annually from 1–5 years post-single vaccination with VLA1553. The secondary objective is to evaluate long-term safety (in terms of new-onset SAEs and follow-up of any ongoing AESIs) 0.5–2 years post-single vaccination with VLA1553. The study is expected to complete by 2026. Initial data from 1 and 2 years’ post-vaccination have shown a sustained seroprotection rate of 99% and 97%, respectively, which was similar in younger and older adults.^73^

#### Safety and immunogenicity of VLA1553 in adolescent populations

An ongoing, prospective, randomized, double-blind, multicentre, pivotal study (NCT04650399) evaluated the safety and immunogenicity of VLA1553 in approximately 750 subjects aged 12 years to < 18 years. Participants were randomized 2:1 to receive VLA1553 or placebo. The primary objective of the study was to evaluate the immunogenicity and safety of the adult dose of VLA1553, 28 days post-single vaccination. Safety and immunogenicity data were collected up to Month 12. The study was completed in February 2024. VLA1553 induced seroprotective titres against CHIKV in 98.8% of adolescents receiving single vaccination and had favourable safety data. These data support submission of an indication expansion into adolescents and will be instrumental to support the licensure application in Brazil, which is currently under review.^74^ The study was funded by the Coalition for Epidemic Preparedness Innovation with the support of the EU Horizon 2020 programme and was carried out in collaboration between Valneva and the Brazilian vaccine manufacturer, Instituto Butantan.

#### Paediatric dose-finding study

A multicentre, prospective, randomized, observer-blinded, three-arm, Phase 2 clinical trial (NCT06106581) is evaluating the full-dose and half-dose formulations of VLA1553 versus control in at least 300 male and female healthy children aged 1–11 years. Participants will be randomized 2:2:1 to the two VLA1553-dose groups (*n =* 120 each) or control (*n =* 60). The trial is expected to complete by July 2025.

#### VLA1553 in patients with human immunodeficiency virus (HIV)

Due to a possibility of systemic infections, live-attenuated vaccines are generally not recommended in patients with a severely suppressed immune system.^75^ However, HIV infection is common in some regions affected by CHIKV.^55^ As VLA1553 is a live-attenuated vaccine, a multicentre, prospective, open-label, uncontrolled, single-arm, Phase 3 clinical trial (NCT06028841) will evaluate the safety, tolerability and immunogenicity of VLA1553 in moderately immunocompromised adult participants (aged ≥18 years) living with HIV, conducted in CHIKV-endemic areas. Approximately 75 male or female participants will be enrolled.

#### Post-marketing studies to confirm efficacy under the accelerated approval pathway

Two key post-marketing effectiveness studies are planned in Phase 4 to be conducted as part of the accelerated approval pathway to support the surrogate of protection in a real-world setting. An observational effectiveness study in a population aged ≥12 years in endemic areas of Brazil after local registration of VLA1553 is expected to begin in 2025 and complete by 2028. The objective of the test-negative case–control study is to confirm the effectiveness of VLA1553 in the prevention of symptomatic laboratory-confirmed chikungunya cases post-single vaccination. Public health infrastructure in Brazil will be utilized to identify cases and controls based on CHIKV-polymerase-chain-reaction testing. A pilot vaccination programme will be implemented in municipalities at high risk for CHIKV outbreaks, selected for the observational effectiveness study. During the pilot vaccination programme, a CHIKV pre-exposure serosurvey, a safety evaluation cohort and a study assessing safety in exposed pregnant women will also be implemented.

A pragmatic randomized controlled effectiveness and safety study in adults from multiple endemic countries will assess the effectiveness of VLA1553 in the prevention of symptomatic laboratory-confirmed chikungunya cases after a single vaccination compared with control participants during the same trial period. Safety evaluation for severe adverse reactions potentially resembling a CHIKV infection and prolonged arthralgia will also be carried out. The study is expected to begin by 2025 and complete in 2029.

## Conclusion

Over three-quarters of the world’s population live in areas at risk of CHIKV and this region is expected to expand. Given the epidemic and outbreak potential, and the travel-associated risk, vaccines such as VLA1553 (IXCHIQ^®^) play an important role in comprehensive strategies to reduce the spread and burden of chikungunya in endemic populations and travellers. Innovative regulatory pathways such as the accelerated approval pathway employed for this vaccine can significantly advance the development of much-needed vaccines against diseases that pose development challenges due to epidemic occurrence or other causes. Although efficacy data are only expected in 2029, implementation of chikungunya vaccination policy and programmes should be considered earlier due to regulatory approval by the FDA and EMA via the surrogate endpoint; as seen with recent issuance of recommendations by the CDC.

Funding: We acknowledge the Coalition for Epidemic Preparedness Innovation (CEPI) and EU Horizon 2020 for partially funding the VLA1553 development programme. This review was designed and funded by Valneva Austria. All authors had access to study data reported in the manuscript, contributed to the drafting and revision of the manuscript, approved the final version and had final responsibility for the decision to submit for publication.

## Supplementary Material

1189315_VLA1553_Clinical_Development_Review_Suppl_Figure_v8_taae123

VLA1553_Review_Supplement_14August24_taae123

## Data Availability

All data are incorporated into the article and its online supplementary material.

## Credit author statement

Lin Chen (Conceptualization [equal], Project administration [supporting], Supervision [equal], Visualization [equal], Writing—original draft [equal], Writing—review & editing [equal]). Andrea Fritzer (Conceptualization [equal], Data curation [equal], Funding acquisition [equal], Investigation [equal], Project administration [supporting], Resources [equal], Supervision [equal], Visualization [equal], Writing—original draft [equal], Writing—review & editing [equal]). Romana Hochreiter (Conceptualization [equal], Data curation [equal], Funding acquisition [equal], Investigation [equal], Project administration [supporting], Resources [equal], Supervision [equal], Visualization [equal], Writing—original draft [equal], Writing—review & editing [equal]). Katrin Dubischar (Conceptualization [equal], Data curation [equal], Funding acquisition [equal], Investigation [equal], Project administration [supporting], Resources [equal], Supervision [equal], Visualization [equal], Writing—original draft [equal], Writing—review & editing [equal]). Stephanie Meyer (Conceptualization [equal], Data curation [equal], Funding acquisition [equal], Investigation [equal], Project administration [lead], Resources [equal], Software [equal], Visualization [equal], Writing—original draft [equal], Writing—review & editing [equal]).
