# Supplementary figures and images for: From bench to clinic: the development of VLA1553/IXCHIQ, a live-attenuated chikungunya vaccine
Source: J Travel Med. 2024 Sep 10;31(7):taae123. doi: 10.1093/jtm/taae123 (PMC11497415; doi:10.1093/jtm/taae123)

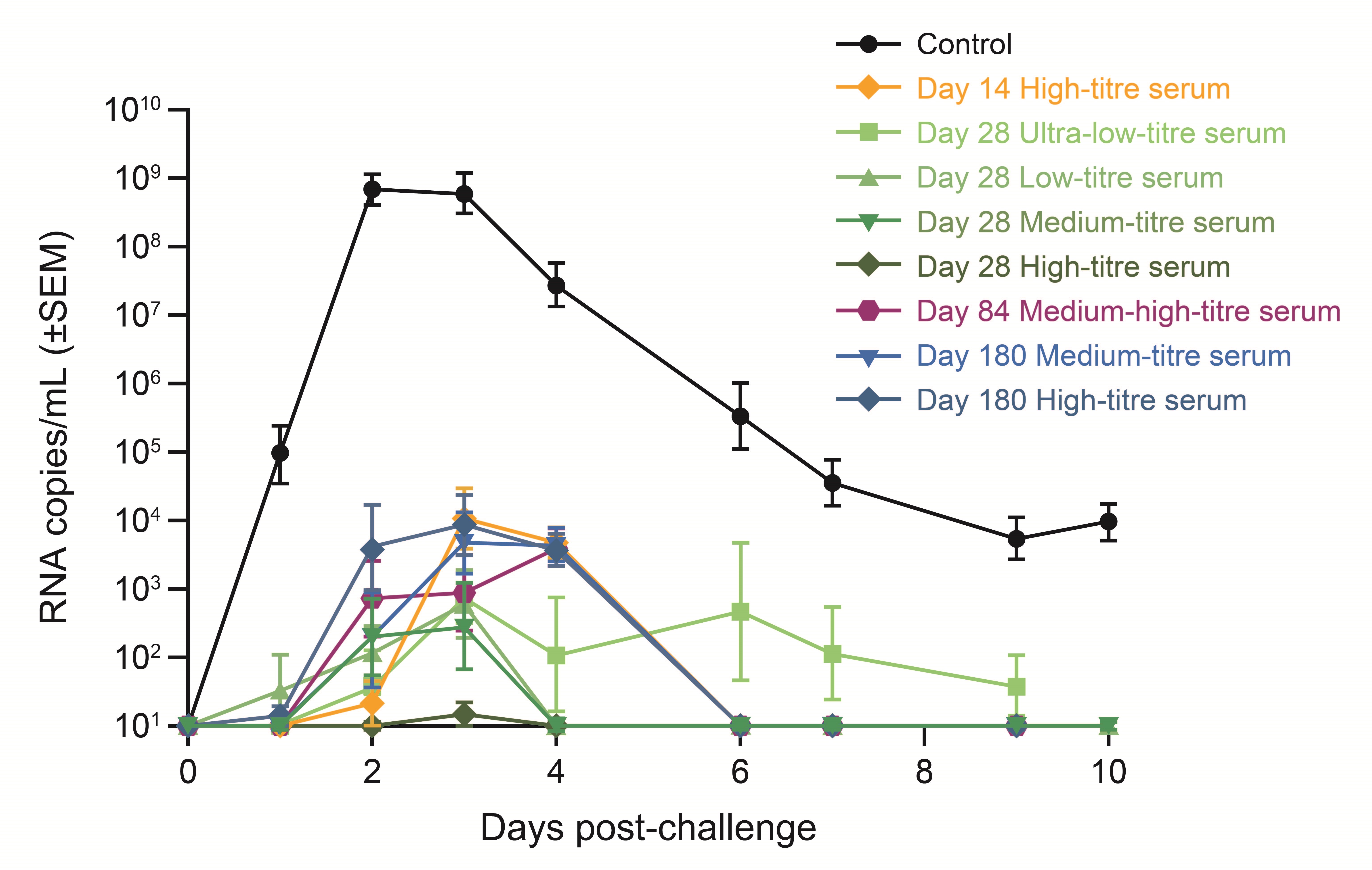

Supplement: 1189315_VLA1553_Clinical_Development_Review_Suppl_Figure_v8_taae123 [file 1189315_vla1553_clinical_development_review_suppl_figure_v8_taae123.jpeg]
