# Supplementary material for: From bench to clinic: the development of VLA1553/IXCHIQ, a live-attenuated chikungunya vaccine
Source: J Travel Med. 2024 Sep 10;31(7):taae123. doi: 10.1093/jtm/taae123 (PMC11497415; doi:10.1093/jtm/taae123)
Supplement: VLA1553_Review_Supplement_14August24_taae123 [file vla1553_review_supplement_14august24_taae123.docx]

**Supplementary information**

**From bench to clinic: the development of VLA1553/IXCHIQ, a live-attenuated chikungunya vaccine**

Lin H. Chen, MD^1,2^, Andrea Fritzer, PhD^3^, Romana Hochreiter, PhD^4^, Katrin Dubischar, MSc^5^, Stéphanie Meyer, PharmD^6^

^1^Department of Medicine, Division of Infectious Diseases and Travel Medicine, Mount Auburn Hospital, Cambridge, MA, USA; ^2^Faculty of Medicine, Harvard Medical School, Boston, MA, USA; ^3^Pre-Clinical Vaccine Development Department, Valneva Austria GmbH, Vienna, Austria; ^4^Clinical Serology Department, Valneva Austria GmbH, Vienna, Austria; ^5^R&D Management, Valneva Austria GmbH, Vienna, Austria; ^6^Corporate Medical Affairs, Valneva SE, Lyon, France

**Serological surrogate endpoint determination: determination of plasma viral load in NHPs by reverse-transcription quantitative polymerase chain reaction^1^**

Plasma viral load in NHPs was determined by reverse-transcription quantitative polymerase chain reaction, which showed that in all animals treated with VLA1553 Phase 1 sera, the viraemia peaks were strongly delayed and reduced by at least 3–5 logs compared with controls that received serum from non-vaccinated humans (Supplementary Figure 1). Sterilizing protection, defined as no detectable CHIKV RNA, was observed in four out of five animals that received the highest titre (in which circulating neutralizing antibody levels after human serum transfer ranged from 82–155 µPRNT_50_) of human post-vaccination serum. No replicating wild-type CHIKV was detected in a TCID_50_ assay in any of the plasma samples from NHPs treated with VLA1553 Phase 1 sera, in contrast to NHPs receiving non-immune sera, which showed detectable infectious CHIKV.

Nearly all animals (39/40, 97.5%) treated with VLA1553 Phase 1 sera showed no significant clinical signs or symptoms of chikungunya disease, except for lymphopenia in one animal. In the control group, all animals (100%) developed fever, lymphopenia and neutrophilia, and displayed a strong inflammatory response after exposure to wild-type CHIKV.

**Supplementary Figure 1.** Viraemia in NHP plasma measured by qPCR.^1^


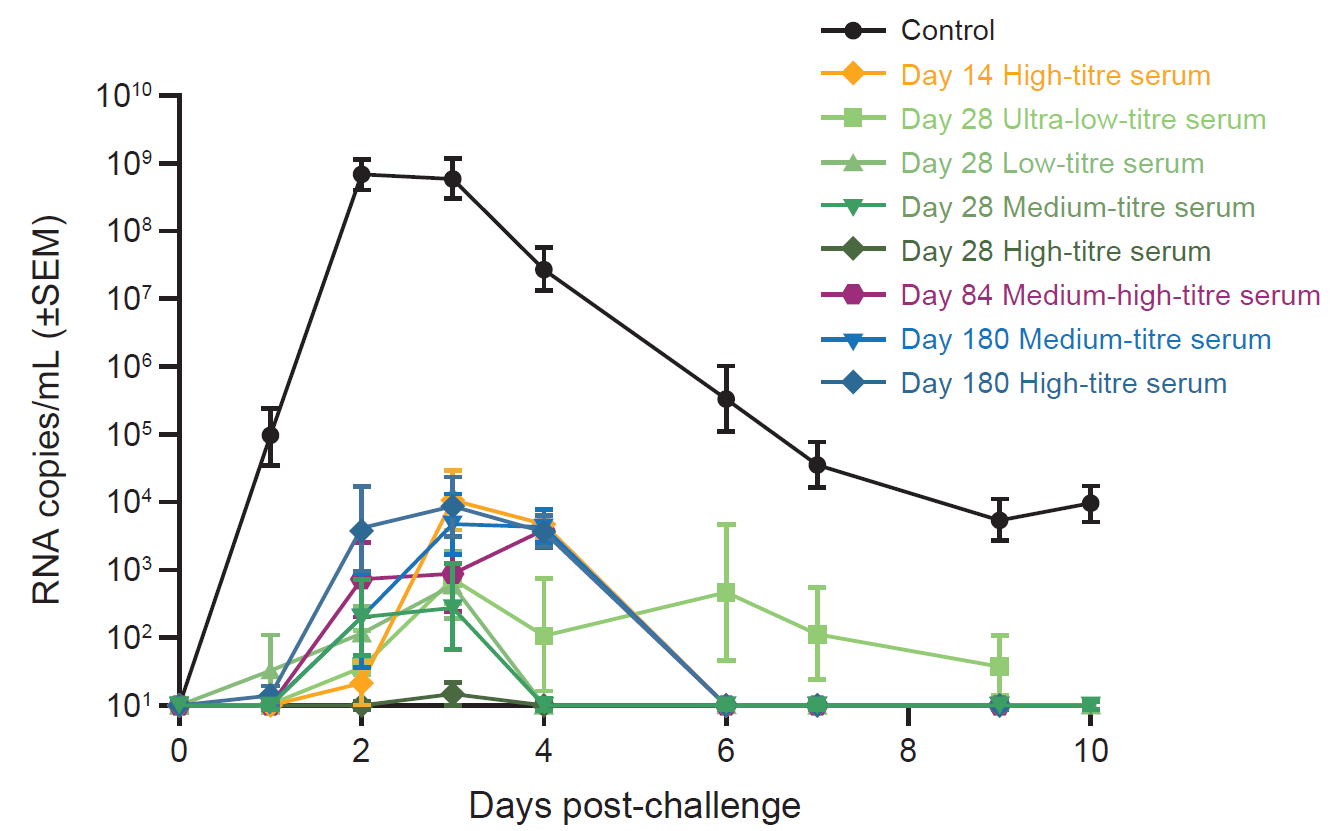


Figure shows mean viraemia ± SEM for control animals who received human non-immune serum and from animals who received human VLA1553 Phase 1 serum obtained at various time-points after vaccination (Day 14–Day 180) with varying μNT_50_ titres ranging from ultra-low to high-titre serum.

NHP, non-human primate; qPCR, quantitative polymerase chain reaction; SEM, standard error of the mean; μNT_50_, micro-neutralization assay, defined as a 50% reduction of cytopathic effect.

Adapted from P Roques, A Fritzer, N Dereuddre-Bosquet, et al. Effectiveness of chikv vaccine vla1553 demonstrated by passive transfer of human sera. JCI Insight 2022; 7(14):e160173, under the terms of the Creative Commons Attribution 4.0 International License (https://creativecommons.org/licenses/by/4.0/deed.en).

**Serological surrogate endpoint determination: Support of the protective antibody threshold by analysis of sera from the sero-epidemiological study^2^**

For all the samples tested, the geometric mean ratio of the values achieved by the two assays (μPRNT_50_/PRNT_80_) was 3.73 (99% CI, 2.86–4.87; Figure 1B in manuscript). The protective PRNT_80_ titre of 10 observed in the Yoon *et al*. study translated to a µPRNT_50_ of 37.3. Applying the highest ratio of μPRNT_50_/PRNT_80_ for an individual sample (13.93) to the protective PRNT_80_ titre of ≥10 derives a μPRNT_50_ titre ≥139.3, which falls within the proposed level of the surrogate of protection, μPRNT_50_ titre ≥150 (Figure 1A in manuscript). The proposed correlate of PRNT_80_ ≥10 in the sero-epidemiological study was shown to be consistent with the surrogate of protection established in this NHP passive transfer study using VLA1553 Phase 1 sera.

# **References**

1 P Roques, A Fritzer, N Dereuddre-Bosquet, et al. Effectiveness of chikv vaccine vla1553 demonstrated by passive transfer of human sera. JCI Insight 2022; 7(14):e160173.

2 IK Yoon, MT Alera, CB Lago, et al. High rate of subclinical chikungunya virus infection and association of neutralizing antibody with protection in a prospective cohort in the philippines. PLoS Negl Trop Dis 2015; 9(5):e0003764.
